# Anti-Osteoporosis Effect of *Perilla frutescens* Leaf Hexane Fraction through Regulating Osteoclast and Osteoblast Differentiation

**DOI:** 10.3390/molecules27030824

**Published:** 2022-01-26

**Authors:** Kanokkarn Phromnoi, Supachai Yodkeeree, Komsak Pintha, Sariya Mapoung, Maitree Suttajit, Chalermpong Saenjum, Pornngarm Dejkriengkraikul

**Affiliations:** 1 Division of Biochemistry, School of Medical Sciences, University of Phayao, Phayao 56000, Thailand; kanokkarn.ph@up.ac.th (K.P.); komsakjo@gmail.com (K.P.); maitree.suttajit@gmail.com (M.S.); 2 Unit of Excellence in Research to Develop Lanna Herbs against Osteoporosis, University of Phayao, Phayao 56000, Thailand; 3 Department of Biochemistry, Faculty of Medicine, Chiang Mai University, Chiang Mai 50200, Thailand; yodkeelee@hotmail.com (S.Y.); srmapoung@gmail.com (S.M.); 4 Center for Research and Development of Natural Products for Health, Chiang Mai University, Chiang Mai 50200, Thailand; 5 Department of Pharmaceutical Sciences, Faculty of Pharmacy, Chiang Mai University, Chiang Mai 50200, Thailand; 6 Cluster of Excellence on Biodiversity-Based Economics and Society (B.BES-CMU), Chiang Mai University, Chiang Mai 50200, Thailand

**Keywords:** *Perilla frutescens*, osteoporosis, osteoclast, osteoblast, RANKL, TNF-α

## Abstract

Osteoporosis is the result of an imbalance in the bone-remodeling process via an increase in osteoclastic activity and a decrease in osteoblastic activity. Our previous studies have shown that *Perilla frutescens* seed meal has anti-osteoclastogenic activity. However, the role of perilla leaf hexane fraction (PLH) in osteoporosis has not yet been investigated and reported. In this study, we aimed to investigate the effects of PLH in osteoclast differentiation and osteogenic potential using cell-based experiments in vitro. From HPLC analysis, we found that PLH contained high luteolin and baicalein. PLH was shown to inhibit RANKL-induced ROS production and tartrate-resistant acid phosphatase (TRAP)-positive multi-nucleated osteoclasts. Moreover, PLH significantly downregulated the RANKL-induced MAPK and NF-κB signaling pathways, leading to the attenuation of NFATc1 and MMP-9 expression. In contrast, PLH enhanced osteoblast function by regulating alkaline phosphatase (ALP) and restoring TNF-α-suppressed osteoblast proliferation and osteogenic potential. Thus, luteolin and baicalein-rich PLH inhibits osteoclast differentiation but promotes the function of osteoblasts. Collectively, our data provide new evidence that suggests that PLH may be a valuable anti-osteoporosis agent.

## 1. Introduction

Bone remodeling is mediated by a balance between osteoblastic bone formation and osteoclastic bone resorption. The disturbance of this balance results in several bone diseases, including arthritis, periodontitis, and osteoporosis [1,2]. Osteoporosis is a major worldwide health problem that affects mainly elderly and postmenopausal women. It leads to an increased risk of fractures and serious health concerns [3,4,5]. The current treatments for osteoporosis are anti-resorptive drugs (such as bisphosphonates, estrogen, selective estrogen receptor modulators, vitamin D, calcium, calcitonin, and denosumab) [6,7,8] and anabolic agents (abaloparatide and teriparatide) [9].

Various clinical studies recommend that osteoporotic treatments should improve the imbalance between bone formation and bone resorption at a cellular level [10]. Recently, there was a hypothesis relating to the possibility that combination therapy with antiresorptive and anabolic drugs would provide even greater benefits than either drug alone [11]. However, patients treated with these inhibitors encountered undesirable negative side effects [12]. Therefore, an identification of natural products is needed. In addition, if it is an antiresorptive and anabolic natural compound in one, it will be beneficial to osteoporosis patients and the health conscious.

Osteoclasts are differentiated from monocyte/macrophage lineage cells in that they are characterized by their multi-nucleated cellular morphology and high tartrate-resistant acid phosphatase (TRAP) expression. Osteoclasts are developed upon induction by a macrophage colony-stimulating factor (M-CSF) and the receptor for activation of nuclear factor-kappa B ligand (RANKL)/osteoprotegerin ligand (OPGL), which are secreted by osteoblasts [13]. The binding of RANKL to the RANK receptor recruits the TNF receptor-associated factor (TRAF) family molecules, which sequentially induces the production of oxidative stress such as reactive oxygen species (ROS) and activates the nuclear factor kappa B (NF-κB) and the mitogen-activated protein kinase (MAPK) pathways [14]. This leads to the activation of transcription factors such as nuclear factor of activated T cells c1 (NFATc1), which stimulates osteoclast development by upregulating downstream osteoclastic target genes, such as matrix metalloproteinase-9 (MMP-9) [15,16].

Osteoblasts are mononuclear cells that are differentiated from mesenchymal stem cells due to the activation of various transcription factors [17]. The Runt-related transcription factor 2 (Runx2) induces the expression of osteogenic genes, including type I collagen (COL1), bone sialoprotein (BSP), osteopontin (OPN), and osteonectin (OSN) especially, and alkaline phosphatase (ALP) and MMP-2, which are involved in the maturation step of osteoblasts [18,19,20,21]. In addition, the differentiation of osteoblasts is regulated by tumor necrosis factor-α (TNF-α), which has an inhibitory influence on bone formation through the inhibition of osteoblast differentiation and the induction of osteoblast apoptosis [22].

Even though novel drugs for osteoporosis treatments that act on different molecular targets of osteoporosis have been developed [23], searching for medicinal plants to rectify the imbalance of bone metabolism is also a great potential alternative approach. Various medicinal plants were used in Thai traditional medicinal remedies for osteoporosis prevention and treatment, such as *Pueraria mirifica*, *Cissus quadrangularis* Linn, *Zingiber montanum*, and *Sesamum indicum* [24,25]. One of the potential plant candidates, *Perilla frutescens* L., has been reported to have osteoclastogenic protection properties and has been used in northern Thai traditional remedies [26].

*P. frutescens* L., known as perilla, or *Nga-Mon* in Thai, is an herb that belongs to the mint family, and is traditionally grown in Thailand and many Asian countries [27]. The perilla leaves are edible and nutritious, and also used as a spice and a food decoration or flavoring [28,29,30]. Several studies indicated that perilla leaf extracts exhibited antioxidant and anti-inflammatory [31,32], antitumor [33,34], anti-diabetic [35], anti-nociceptive [36], anti-tuberculosis, and antimicrobial [37] activities. Recently, phytochemical studies have shown that water extract of perilla leaves contains rosmarinic acid, luteolin, baicalin, rutin, catechin, apigenin, gallic acid, chlorogenic acid, l-epicatechin, baicalein, and other flavonoids [30,38]. Nevertheless, phytochemical compounds in the perilla leaf hexane fraction (PLH) had never been reported. Therefore, in this study, we evaluated the active compounds in PLH.

Numerous flavonoids, including rutin, hesperidine, genistein, daidzein, and anthocyanins, have demonstrated beneficial effects for bone health in various targets and mechanisms of osteoporosis prevention and treatment [39]. Interestingly, a synthetic flavonoid, luteolin, has been shown to inhibit RANKL-induced osteoclastogenesis through the suppression of activation of activating transcription factor 2 (ATF2), downstream of p38 MAPK and NFATc1 expression [40]. Moreover, purified luteolin promoted osteoblastic proliferation and differentiation by attenuating oxidative stress and regulating the ratio of osteoprotegerin/RANKL and induced various osteogenic markers [41]. It is reported that synthetic baicalein inhibits bone resorption by inducing the apoptosis of mature osteoclasts [42], and that purified baicalein promotes osteoblastic differentiation by inducing the transcripts and protein expressions of OSX, COL-1, and RUNX-2 through MAPK and the Wnt/β-catenin pathway [43]. Furthermore, our previous studies found that rosmarinic acid-enriched perilla seed meal showed anti-osteoclastogenesis in the RAW264.7 cell line [26]. However, the role of luteolin and baicalein-rich PLH in osteoporosis has not been investigated and reported yet.

The aim of this study was to investigate the effect of PLH on osteoclastogenic activity and osteogenic potential. We established the effect of PLH on RANKL-induced osteoclast differentiation and the underlying mechanisms. Moreover, the effects of PLH on osteogenic potential without or with TNF-α treatment were investigated by measuring the cell proliferation, ALP, and MMP-2 activities.

## 2. Results

### 2.1. Determination of Active Compounds in PLH

**Figure 1:**
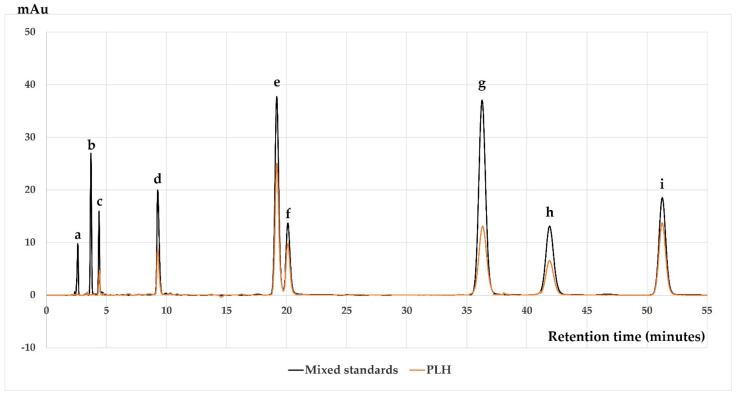
HPLC chromatograms of mixed standard and PLH. The peaks reveal (**a**) gallic acid, (**b**) caffeic acid, (**c**) rutin, (**d**) rosmarinic acid, (**e**) luteolin, (**f**) quercetin, (**g**) apigenin, (**h**) kaempferol, and (**i**) baicalein. The mobile phase consisted of 30% acetonitrile in 0.1% acetic acid and de-ionized water at a flow rate of 1.0 mL/min. The content peaks were detected by a UV detector at 325 nm.

The quantification mount of phytochemical constituents of PLH was analyzed by reversed-phase HPLC. The PLH compared to the mixed standard demonstrated that luteolin and baicalein were dominant compounds, followed by apigenin, kaempferol, quercetin, rosmarinic acid, and rutin as shown in Figure 1 and Table 1. The separation of mixed compounds in PLH from the hydrophobic stationary phase HLPC column corresponded to the Log *p*-value of each compound, as shown in Table 1.

### 2.2. Effect of PLH on Cell Viability

**Figure 2:**
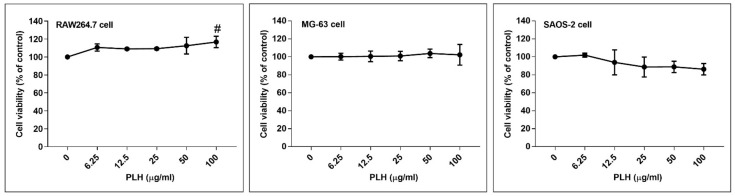
Viability of RAW264.7, MG-63, and SAOS-2 cells incubated with various concentrations of PLH using MTT assay. Each value is the mean ± SD of three independent experiments. # *p* < 0.05 vs. control (0).

The cytotoxicity effects of PLH against the macrophage, RAW264.7 cells and osteoblast-like, MG-63 and SAOS-2 cells were examined by MTT assay. As shown in Figure 2, there was no significant decline in cell viability at concentrations ranging lower than 100 μg/mL at 48 h treatment compared to untreated cells. At least three non-cytotoxic dosages of the PLH (12.5, 25, and 50 μg/mL) were selected for further experiments.

### 2.3. Effect of PLH on ROS Production in RAW264.7 Cells

**Figure 3:**
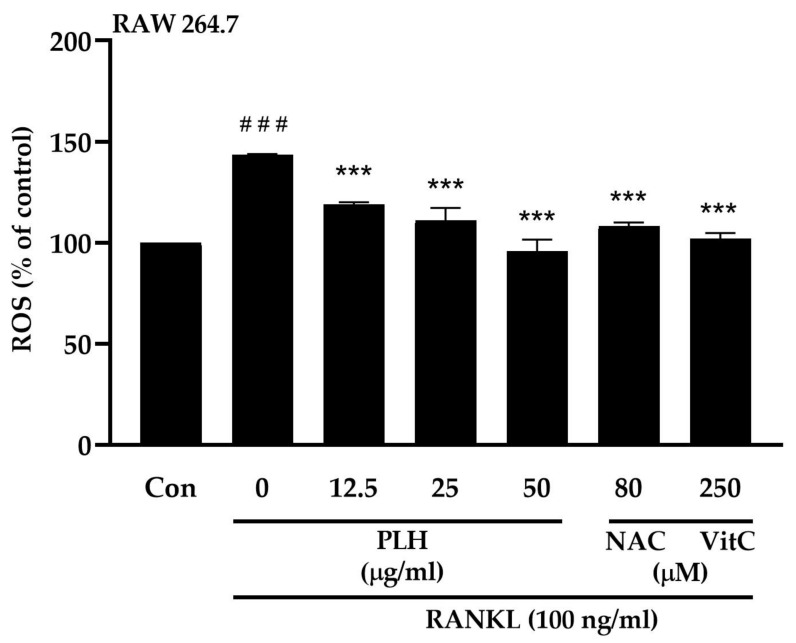
RANKL-induced ROS production by PLH treatment. Untreated cells were used as a negative control (Con). N-acetylcysteine 80 μM (NAC) and vitamin C 250 μM (Vit C) were used as positive controls. Each value is the mean ± SD of three independent experiments, ### *p* < 0.001 vs. control (Con), *** *p* < 0.001 vs. RANKL treatment (0).

Monocyte-/macrophage-like cells, RAW264.7 cells, can differentiate into osteoclasts in the presence of RANKL. They are also evaluated as a great model for in vitro osteoclast differentiation studies [44,45]. RANKL induces intracellular ROS generation, which leads to the activation of osteoclast differentiation [35,46,47]. Therefore, the effect of PLH on RANKL-induced ROS production was determined. The results show that ROS production was enhanced through RANKL stimulation up to 143.33 ± 0.17% of the control. However, the induced formation of intracellular ROS was significantly attenuated by the treatment of PLH, the same as NAC and vitamin C (*p* < 0.001) (Figure 3). Thus, we can hypothesize that the neutralization of ROS by PLH may subsequently prevent osteoporosis.

### 2.4. Effects of PLH on RANKL-Mediated TRAP-Positive Osteoclast-Like Cell Formation In Vitro

**Figure 4:**
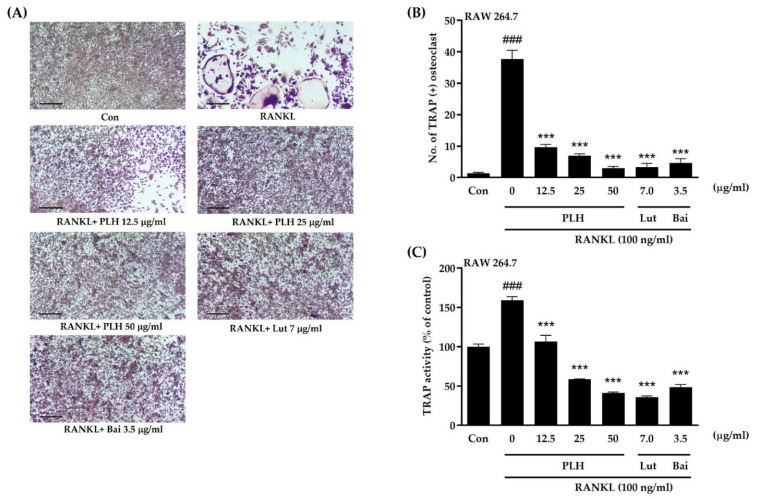
RANKL-induced TRAP-positive osteoclast-like cell formation by PLH treatment. (**A**) Osteoclast differentiation: Multinucleated osteoclasts were visualized in 100× magnification under light microphotography. Scale bars, 100 mm. (**B**) TRAP-positive multinucleated cells were counted as osteoclasts. (**C**) TRAP activity was measured using the TRAP solution assay. Luteolin (Lut) and baicalein (Bai) were active compounds of PLH. Each value is the mean ± SD of three independent experiments. ### *p* < 0.001 vs. control (Con), *** *p* < 0.001 vs. RANKL treatment (0).

In our study, RAW264.7 cells were cultured in the presence of 100 ng/mL RANKL and various concentrations of PLH (0, 12.5, 25, and 50 μg/mL). After six days of incubation, the multinucleated TRAP-positive cells were stained and visualized through microscopy. TRAP activity was determined using a TRAP solution assay. Luteolin and baicalein, key active ingredients of PLH, were parallel evaluated in the same experiment. Figure 4A,B illustrates that RANKL treatment induced the differentiation of RAW264.7 cells into osteoclast-like multinucleated (three or more nuclei) cells.

In contrast, when the cells were treated with PLH, the number of multinucleated cells was remarkably suppressed in a dose-dependent manner. Luteolin at 7 µg/mL and baicalein at 3.5 µg/mL, calculated to be equivalent to 50 μg/mL of PLH, similarly suppressed RANKL-induced osteoclast differentiation. TRAP activity was also quantitatively examined: It was found that the activity was increased up to 159.15 ± 7.9% after treatment with RANKL compared to the control. PLH significantly inhibited RANKL-induced TRAP activity in a dose-dependent manner, similar to luteolin and baicalein treatment (*p* < 0.001) (Figure 4C). These results indicate that luteolin and baicalein-enriched PLH can exhibit a potent inhibitory effect on RANKL-induced osteoclast formation.

### 2.5. Effect of PLH on Osteoclastic-Specific Protein Expression 

**Figure 5:**
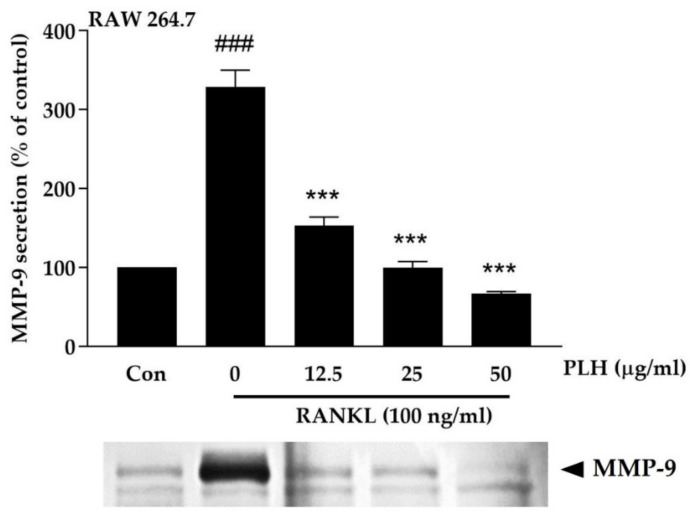
RANKL-induced MMP-9 expression by PLH treatment. RAW264.7 cells were co-treated with RANKL and PLH for 72 h. Culture supernatants were collected, and the secretion of MMP-9 was analyzed using gelatin zymography. Each value is the mean ± SD of three independent experiments. ### *p* < 0.001 vs. control (Con), *** *p* < 0.001 vs. RANKL treatment (0).

MMP-9 is the most abundant gelatinolytic enzyme in osteoclasts, which has a significant role in bone matrix degradation and resorption [48,49]. MMP-9 can be used as one alternative way to measure resorption activity, as previously described elsewhere [50,51]. Moreover, MMP-9 secretion can be induced by RANKL after long-term incubation [52,53]. To further identify the inhibitory effect of PLH on RANKL-induced osteoclastogenesis, we investigated the expression of this osteoclast-specific protein marker, MMP-9, using gelatin zymography assays. Figure 5 illustrates that MMP-9 activity was enhanced up to 328.83 ± 36.88% after RANKL incubation for three days. However, the RANKL-induced MMP-9 levels was significantly decreased after PLH treatment in a dose-dependent manner (*p* < 0.001). Collectively, these results suggested that PLH suppressed osteoclast differentiation and function.

### 2.6. Effects of PLH on RANKL-Induced NF-κB, MAPK, and NFATc1 during Osteoclast Differentiation

**Figure 6:**
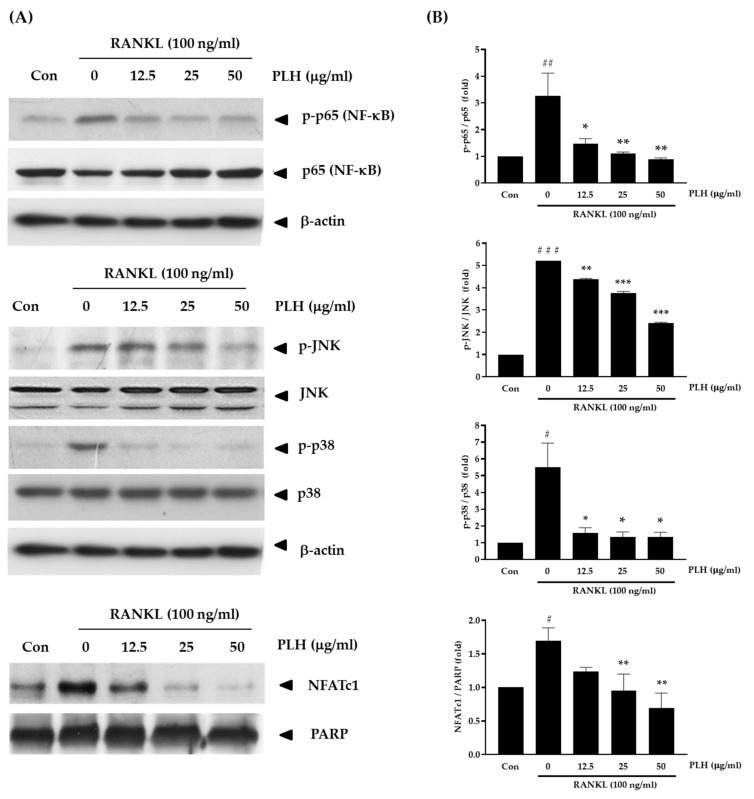
RANKL-induced NF-κB, MAPK, and NFATc1 signaling by PLH treatment. RAW 264.7 cells were pretreated with PLH (0, 12.5, 25, and 50 µg/mL) for 12 h and then exposed to RANKL (100 ng/mL) for 10 min. The whole cell extract was used to determine phosphorylation levels of NF-κB, JNK, and p38. The nuclear extracts were prepared and NFATc1 expression was analyzed. (**A**) Protein expression of NF-κB, MAPKs, and NFATc1 was measured using the Western blot method. (**B**) The expression of p-p65, p-JNK, p-p38, and NFATc1 was normalized to total p65, JNK, p38, and PARP, respectively. Each value is the mean ± SD of three independent experiments. ### *p* < 0.001; ## *p* < 0.01; # *p* < 0.05 vs. control (Con), *** *p* < 0.001; ** *p* < 0.01; * *p* < 0.05 vs. RANKL treatment (0).

The combination of RANKL and RANK leads to the activation of downstream cell-signaling pathways, such as the NF-κB and MAPK pathways [54,55,56,57], which, in turn, results in the activation of the critical osteoclast differentiation-related transcription factors, such as NFATc1. This leads to the expression of downstream targets, including TRAP, cathepsin K, and MMP-9. Thus, agents suppressing these signals may inhibit osteoclastogenesis and bone loss. It would be interesting to discover whether PLH can inhibit osteoporosis through the NF-κB, MAPK, and NFATc1 pathways. Due to the phosphorylation of NF-κB (p50/p65), which plays a significant role in the stimulation of its pathway, we focused on NF-κB phosphorylation using Western blotting analysis. Figure 6A,B demonstrates that the phosphorylation of p65 was evidently increased after RANKL stimulation. However, the PLH treatments at various concentrations remarkably impaired the RANKL-induced signaling molecules to the control level. As the phosphorylation of p38 and JNK also plays a vital role in RANKL-induced osteoclastogenesis [58], the inhibitory effect of PLH on p38 and JNK activation was also studied. The results show that the phosphorylation of p38 and JNK immensely increased when treated with RANKL. In contrast, the PLH treatment significantly downregulated the phosphorylation of p38 and JNK. Additionally, similar to the results of NF-κB and MAPK, PLH can abrogate RANKL-induced NFATc1.

### 2.7. Effect of PLH on Osteogenic Potential

**Figure 7:**
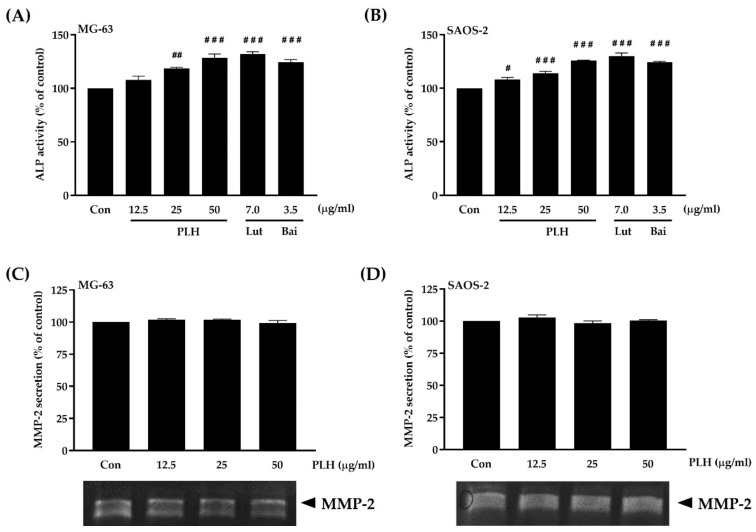
ALP activity and MMP-2 expression in osteoblast-like cells by PLH treatment. (**A**) MG-63- and (**B**) SAOS-2-treated cells were tested for ALP using colorimetric analysis. (**C**) MG-63 and (**D**) SAOS-2 culture supernatants were collected and analyzed for MMP-2 secretion by gelatin zymography. Luteolin (Lut) and baicalein (Bai) were active compounds of PLH. Each value is the mean ± SD of three independent experiments. ### *p* < 0.001; ## *p* < 0.01; # *p* < 0.05 vs. control (Con).

Besides osteoclastogenesis, we also studied osteoblast function using MG-63 and SAOS-2 cells [59,60,61,62,63] by measuring the activities of ALP and MMP-2 as important markers for the early and late differentiation of osteoblast cells, respectively [64]. ALP can hydrolyze inorganic pyrophosphate, which is a naturally occurring inhibitor of mineralization [65], whereas MMP-2 can shed the immune costimulatory molecule B7-H3 from the cell membrane of osteoblasts, leading to bone mineralization [66]. Figure 7A,B shows the increase in ALP activity in both MG-63 and SAOS-2 osteoblast cells after PLH treatment for 72 h, consistent with luteolin and baicalein treatment, which are the main compounds in PLH. In contrast, Figure 7C,D shows that PLH did not affect MMP-2 levels in either MG-63 or SAOS-2 cells compared to the control. Based on these results, PLH is able to induce the differentiation of early-stage osteoblasts through ALP activation and is not associated with MMP-2.

### 2.8. Effects of PLH on TNF-α-Suppressed Osteogenic Potential

**Figure 8:**
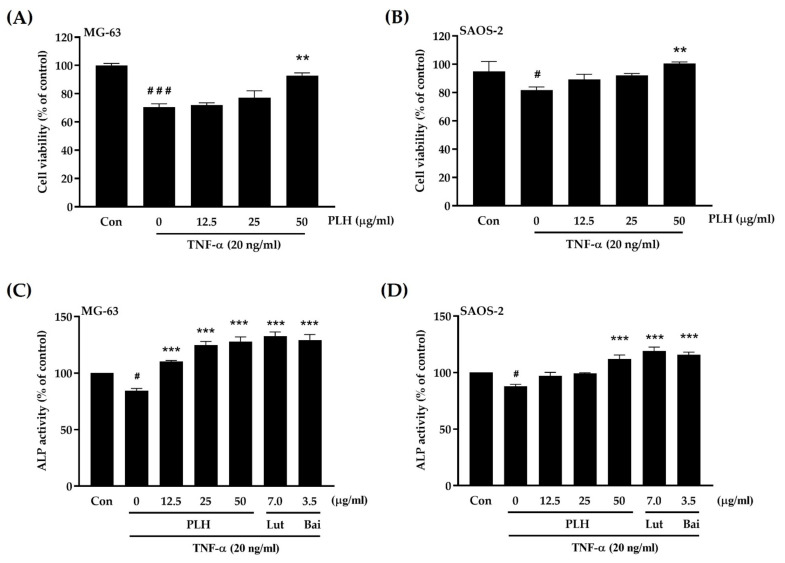
PLH restored TNF-α-suppressed osteogenic potential. Cells were co-treated with TNF-α (20 ng/mL) and various concentrations of PLH (0–50 μg/mL) for 48 h (cell proliferation) and 72 h (ALP activity). (**A**) MG-63- and (**B**) SAOS-2-treated cells were measured for viability using MTT assay. (**C**) MG-63- and (**D**) SAOS-2-treated cells were tested for ALP using colorimetric analysis. Each value is the mean ± SD of three independent experiments. ### *p* < 0.001; # *p* < 0.05 vs. control (Con), *** *p* < 0.001; ** *p* < 0.01 vs. TNF-α treatment (0).

The activity of osteoblasts is regulated by the inflammatory cytokine TNF-α, which induces osteoblast apoptosis and inhibits osteoblast differentiation [67,68,69]. Therefore, any compounds that inhibit TNF-α-suppressed cell viability and differentiation may prevent bone diseases. The osteoprotective effects of PLH on TNF-α-treated osteoblasts were elucidated using MTT and ALP activity assays. As demonstrated in Figure 8A,B, PLH significantly restores TNF-α-suppressed osteoblast-like cell viability.

In addition to TNF decreased cell viability, TNF-α treatment also resulted in a significant reduction in ALP activity in MG-63 and SAOS-2 cells (Figure 8C,D). The reduction activity was ameliorated in response to PLH treatments, including luteolin and baicalein treatments, which were used as positive controls, suggesting that PLH was able to significantly protect osteoblasts from TNF-α-suppressed osteogenic potential. Based on these results, PLH is able to induce osteoblasts.

## 3. Discussion

Osteoporosis has been specified as a metabolic disorder characterized by an imbalance in the bone-remodeling process. The resorption of bone is increased without adequate new bone formation, inducing osteoblast apoptosis and suppression of osteoclast apoptosis [70]. As a result of the high costs and side effects of anti-osteoporotic or anabolic chemical agents, the search for natural products that would allow for more affordable treatment of this disease, with more marginal side effects, has become essential [6]. The focus of this study was perilla leaves, less commonly eaten than perilla seeds, which we suggest may have value as an alternative food in our diets.

After the solvent partition isolation technique was used, we evaluated the number of active compounds in the other fraction by using HPLC. We found that each fraction contained different bioactive compounds. The main bioactive compounds contained in leaf water fraction were rutin, caffeic acid, and gallic acid. The leaf ethyl acetate fraction indicates that rosmarinic acid was the major active compound. Meanwhile, luteolin and baicalein were the major active compounds in the leaf hexane fraction. The leaf hexane fraction showed the highest inhibitory effect on ROS production compared to the water, dichloromethane, and ethyl acetate fractions (data not shown). Therefore, we next focused on the hexane fraction. The PLH contains the phytochemical compounds listed in Table 1, and most compounds can be obtained from the hexane fraction. This is partitioned from crude ethanolic extract, which offers a greater yield than those partitioned from water extract, so it is more advantageous.

Phytochemical constituents from the HPLC analysis showed that the PLH contained high amounts of luteolin and baicalein but less apigenin, kaempferol, quercetin, rosmarinic acid, and rutin. Importantly, the Log *p*-value is the logarithm of the partition coefficient between the concentrations of a solute in immiscible binary-phase solvents when one of the solvents is water and the other is octanol, which measures for the lipophilicity or hydrophobicity of each compound. As shown in Table 1, baicalein had the highest Log *p*, followed by kaempferol, apigenin, luteolin, quercetin, rosmarinic acid, caffeic acid, and rutin. According to the procedure of PLH, which is partitioned from crude ethanolic extract, luteolin and baicalein are the major components. Pintha et al. reported that luteolin was a major constituent in crude perilla leaf ethanolic extract [33]. Additionally, Wang et al. reported that rosmarinic acid, rutin, luteolin, and catechin were the dominant components in the ethyl acetate fraction that was partitioned from crude ethanolic leaf extract [71]. In the current study, we propose that luteolin and baicalein were distributed from the crude ethanolic extract, which was dissolved in deionized water, to hexane due to a high value of Log *p* during the solvent partition process.

In addition, studies done on the active ingredients of PLH, luteolin and baicalin, have shown that luteolin can actually inhibit osteoclast differentiation and defense against bone loss, and help in the prevention of osteoporosis [72]. Luteolin also suppresses the mitochondrial apoptosis of osteoblasts via inhibition of STAT1 activity [73]. Moreover, baicalin was shown to be therapeutic for osteoclast-related bone diseases through the inhibition of the NF-κB and ERK pathways [74]. Baicalein can stimulate osteoblast differentiation via the activation of the mTORC1 signaling pathways, including the P-S6K1 and P-4E/BP1 transcription factors [75].

RANKL signaling initiates enhanced osteoclast formation and bone resorption. As such, inhibiting osteoclast formation could be a valuable form of treatment for pathological bone loss [76]. Physiologically, ROS plays an important role in the remodeling process, whereby overproduction of ROS by osteoclasts accelerates bone resorption [77]. Our study demonstrated a high production rate of ROS in RAW264.7 cells after RANKL stimulation was scavenged using PLH treatment. The strong ROS inhibition of hexane extract may suggest that hexane, which has a polarity index amount of 0 (PI = 0), was able to extract lipophilic compounds that can diffuse easily through the lipid-bilayer membrane [78]. Thus, lipophilic compounds may enhance cellular uptake and cellular antioxidant effects, such as ROS generation [79]. It is known that RANKL stimulation upregulates the expression of TRAP and MMP-9, important proteins in osteoclasts, to degrade the organic bone matrix [80,81,82]. Our results indicate that PLH inhibited RANKL-induced osteoclast formation and differentiation from macrophage to multinucleated cells by TRAP staining and colorimetric method, consistent with luteolin and baicalein treatment; these are the main active compounds of PLH. Moreover, the results from gelatin zymography further reveal that the anti-resorption effects of PLH were accompanied by diminished expression levels of MMP-9.

NF-κB, MAPK, and NFATc1 signaling pathways are known to play a significant role in osteoclast differentiation. Binding of RANKL to RANK leads to the recruitment of TRAP6 [83] and activates NF-κB and MAPK [84], leading to the activation of NFATc1, which in turn results in the expression of downstream targets, including TRAP and MMP-9 [85]. A specific inhibitor of p38 MAPK suppressed RANKL-mediated osteoclast differentiation in RAW 264.7 cells [86], and osteoclast precursor cells derived from JNK1-lacking mice exhibited reduced osteoclast differentiation [87]. p38 affects early osteoclastogenesis and plays an important role in the expression of cathepsin K [88], which JNK-deficient rats are unable to differentiate into osteoclasts [87]. RANKL also activates the classical NF-κB pathway after degradation of IκB through a ubiquitin/proteasome pathway. NF-κB is subsequently phosphorylated and translocated from the cytoplasm into the nucleus [89]. A previous study demonstrated that selective inhibition of NF-κB activation blocked osteoclastogenesis and prevented inflammatory bone destruction in vivo and in vitro [56]. To investigate the molecular mechanisms associated with the inhibitory effects of PLH, Western blotting was used. The present study confirmed that PLH suppressed osteoclast differentiation by inhibition of the phosphorylation of JNK, p38, and p65, as well as the expression of NFATc1, and subsequently reduced the expression of TRAP and MMP-9.

Oxidative stress and ROS are also fundamental in osteoblasts, and many previous studies verified the bone anabolic activities of natural products by using these targets [90,91,92,93]. Additionally, osteoblast studies also have also looked at alternative pathways such as Wnt/β-catenin to investigate the impairment or the amelioration of osteogenic potential [94,95,96].

However, in our preliminary studies of osteogenic potential, we also investigated how the regulation of ALP and MMP-2 plays a significant role in the early and late differentiation of osteoblast cells, respectively. Therefore, the focus was on whether PLH could moderate ALP and MMP-2 activation. Our findings showed that PLH significantly increased ALP activity both with and without TNF-α stimulation. However, PLH did not affect the MMP-2 levels. Moreover, PLH could restore TNF-α-suppressed osteogenic potential, maybe as a result of its anti-apoptotic effect. These results suggest that the potential of PLH may be induced pro-anabolic activities. The physiopathological framework, including the intracellular pathway of osteogenic molecules on PLH, requires further study.

## 4. Materials and Methods

### 4.1. Reagents and Chemicals

Recombinant mouse soluble RANKL was obtained from R&D Systems (Minneapolis, MN, USA). Dulbecco’s Modified Eagle Medium (DMEM), α-Modified Eagle Medium (α-MEM), and DMEM/F-12 medium was acquired from Invitrogen (Carlsbad, CA, USA). Fetal bovine serum (FBS) and penicillin–streptomycin were purchased from Thermo Fisher Scientific (Burlington, ON, Canada). The TRAP staining kit, RA, luteolin, and apigenin were purchased from Sigma-Aldrich (St. Louis, MO, USA). Specific antibodies and goat anti-rabbit IgG horseradish peroxidase (HRP) secondary antibodies were obtained from Cell Signaling Technology (Danvers, MA, USA).

### 4.2. Preparation of Perilla Leaf Hexane Fraction (PLH)

Perilla leaves were collected from the Wiang-Sa district, Nan province, Thailand. The voucher specimen code is QSBG-K2, prepared by K. Pintha and P. Tantipaiboonwong, and was certified by the Queen Sirikit Botanic Garden Herbarium, Chiang Mai, Thailand.

The ethanolic crude extract of perilla leaf (LE) was fractionated with hexane and solvent was removed under reduced pressure and then lyophilized to obtain perilla leaf hexane fraction (PLH). The dried fraction was stored at 20 °C and suspended in dimethyl sulfoxide (DMSO) before use.

### 4.3. Chromatographic Analysis for Phenolic and Flavonoid Compounds

A reversed-phase HPLC, Agilent 1200, equipped with the multi-wavelength and fluorescence detectors, was used to separate, identify, and quantify phenolic and flavonoid compounds as previously described [26]. The assay was carried out using a SymmetryShield^®^ RP18 column (4.6 mm × 250 mm, 5 µm particle diameters, Waters Co., Ltd., Milford, MA, USA). The mobile phase consisted of 30% acetonitrile in 0.1% acetic acid and de-ionized water at a flow rate of 1.0 mL/min. The content peaks were detected using a UV detector at 325 nm. The amounts of each detected compound in the samples were calculated and expressed as mg/g extract.

### 4.4. Cell Cultures

Mouse macrophage cell line RAW 264.7, human osteoblast-like MG-63 cells, and human osteoblast-like SAOS-2 cells were obtained from American Type Culture Collection (Manassas, VA, USA). RAW 264.7 and MG-63 cells were cultured in DMEM containing 10% heat-inactivated FBS and 100 U/mL penicillin–streptomycin under 5% CO_2_ at 37 °C. SAOS-2 cell were cultured in DMEM/F-12 containing 10% heat-inactivated FBS and 100 U/mL penicillin–streptomycin under 5% CO_2_ at 37 °C.

### 4.5. Cell Viability Assay

Cell viability was measured by MTT assays as previously described [97], with slight modifications. In brief, the cells were cultured in a 96-well plate with a density of 5 × 10^3^ cells/well. Then, the cells were cultured with various concentrations of PLH (0–100 μg/mL) for 48 h. Thereafter, MTT was added to each well and incubated at 37 °C for 4 h. The medium was then removed. DMSO was added to each well, and the color intensity was measured at 540 and 630 nm using a Cytation 5 multi-mode microplate reader and Gen5 software (Agilent, Santa Clara, CA, USA).

### 4.6. Measurement of Intracellular ROS

The inhibitory effect of PLH on intracellular ROS production was investigated through the measurement of the oxidation of 2′,7′-dichlorodihydrofluorescein diacetate (DCFH-DA) to fluorescent 2′,7′-dichlorofluorescein (DCF) as previously described [98,99], with slight modifications. RAW264.7 cells (1 × 10^4^ cells/mL) were cultured in a 96-well culture plate and pre-treated with 0–50 µg/mL of the fraction for 1 h, followed by treatment with 100 ng/mL RANKL for 1 h. Then, 40 µM of DCFH-DA solution was added. Following incubation at 37 °C and 5% CO_2_ for 30 min, the green fluorescence intensity was measured by fluorescent microplate reader at excitation and emission wavelengths of 480 and 525 nm, respectively. N-acetyl cysteine (80 µM) and vitamin C (250 µM) were used as positive antioxidant controls.

### 4.7. Differentiation of RAW 264.7 Cells into Osteoclast-like Cells

RAW264.7 cells were differentiated into osteoclasts by RANKL. RAW 264.7 cells (5.0 × 10^3^ cells/well) were seeded into a 96-well plate with a DMEM culture medium for 24 h. Then, the medium was replaced with a differentiation medium (α-MEM) containing 100 ng/mL RANKL and the samples (PLH, luteolin, and baicalein) for 6 days, with a replacement of fresh medium every 3 days. Osteoclasts were stained for TRAP using a commercial leukocyte acid phosphatase kit (Sigma-Aldrich, St. Louis, MO, USA) following the manufacturer’s protocol. The TRAP-positive cells with three or more nuclei were counted as osteoclasts and visualized under an inverted microscope (Olympus, Tokyo, Japan) [26].

### 4.8. Tartrate-Resistant Acid Phosphatase (TRAP) Activity Assay

Colorimetric analysis for TRAP activity was based on the ability of the phosphatases to catalyze the hydrolysis of the *p*-nitrophenyl phosphate (*p*-NPP) to a chromogenic product, *p*-nitrophenol (*p*-NP), with absorbance at 405 nm. The assay was measured as previously defined [26], with some modifications. Briefly, the treated cells were lysed with 0.1% Triton X-100 and incubated for more than 2 h at −80 °C. The lysate was defrosted at 37 °C. Subsequently, 25 µL of the substrate solution (1 mg/mL PNPP) was added to 25 µL of the lysate and incubated for 4 h at 37 °C. The reaction was stopped with 50 μL of 0.5 M NaOH, and the absorbance was measured at 405 nm with a microplate reader. *p*-NP was used as a standard.

### 4.9. Determination of Matrix Metalloproteinase-9 (MMP-9) Activity

MMP activity in cell culture supernatants was measured using gelatin zymography as previously described [100], with slight modifications. Culture supernatants were subjected to electrophoresis in a 10% polyacrylamide gel containing 1% gelatin. After electrophoresis, gels were soaked in 2.5% *v/v* Triton X-100 for 30 min, 2 times, and then they were incubated at 37 °C and kept in an activating buffer (50 mM Tris-HCl, 200 mM NaCl, and 10 mM CaCl_2_) at pH 7.4 for 48 h. Bands were stained with Coomassie Brilliant Blue R and de-stained for 2 h at room temperature in a solution (10% acetic acid in 30% methanol). Images were taken using a Bio-Rad Gel Doc XR system (Hercules, CA, USA). Clear bands corresponding to gelatinolytic activity were quantitated using ImageJ software (NIH).

### 4.10. Western Blotting Analysis

RAW264.7 cells (1 × 10^6^ cells/well) were cultured in a 12-well plate, pretreated with various concentrations of PLH (0–50 µg/mL) for 12 h, and stimulated with RANKL (100 ng/mL) for 10 min. Whole cell extracts were prepared with RIPA lysis buffer. Nuclear fractions were prepared using the nuclear extraction buffer. Protein concentration was determined using the Bradford Protein Assay Kit (Bio Basic Inc., Markham, ON, Canada). Equal amounts of protein were subjected to sodium dodecyl sulfate polyacrylamide gel electrophoresis (SDS-PAGE), followed by transfer to nitrocellulose membranes. After blocking with 5% BSA, the bands were probed with specific primary antibodies and incubated at 4 °C overnight. HRP-conjugated secondary antibodies were then added. After incubation, the targeted proteins were visualized by enhanced chemiluminescence (ECL) reagents (Bio-Rad, Hercules, CA, USA). Images were exposed to the X-ray film (GE Healthcare Ltd., Chicago, IL, USA). PARP and β-actin were used as the internal reference.

### 4.11. Alkaline Phosphatase (ALP) Activity Assay

Osteogenic potential has been studied using osteoblast-like cell lines, including MG-63 and SAOS-2 cells [101,102]. ALP activity was measured via the hydrolysis of *p*-NPP. After treating the cells for 72 h, 0.1% Triton X-100 was added to each well and incubated for more than 2 h at −80 °C. The lysate was defrosted at 37 °C. Then, 25 µL of the substrate solution (1 mg/mL *p*-PNPP) was added to 25 µL of the lysate and incubated for 4 h at 37 °C. The reaction was stopped with 50 µL of 0.5 M NaOH and the absorbance was measured at 405 nm with a microplate reader. *p*-NP was used as a standard.

### 4.12. Statistical Analysis

All experiments were performed in triplicate and data are presented as mean ± standard deviation (SD). The results were statistically analyzed using GraphPad Prism 9 Software (La Jolla, CA, USA). # *p* < 0.05, ## *p* < 0.01, and ### *p* < 0.001 vs. control and * *p* < 0.05, ** *p* < 0.01, and *** *p* < 0.001 vs. RANKL or TNF-α treatment were used as the significant difference.

## 5. Conclusions

This is a unique report, because it shows the effects of luteolin- and baicalein-rich PLH on bone resorption and formation. We demonstrated that PLH regulates osteoclast by blocking RANKL-induced ROS production, NF-κB, MAPK phosphorylation, NFATc1 expression, TRAP activity, and MMP-9 secretion. PLH also promotes the function of osteoblasts by increasing ALP activity and restoring TNF-α-suppressed osteogenic potential. Therefore, PLH-targeting treatments for osteoporosis not only suppressed bone resorption but also promoted bone formation. It can be suggested that PLH might be a potential preventive and therapeutic agent for osteoporosis disease. Further studies will be required to confirm the efficacy of PLH in treating bone disease conditions. Such studies should employ a broader physiopathological framework for analysis of the intracellular pathways of osteogenic molecules in vivo and be carried out in a clinical setting.

## Figures and Tables

**Table 1:** The HPLC analysis and Log *p*-values of the phytochemical constituents of PLH, ranking from the highest to the lowest amount.

Compounds	Amount	Log *p*
	(mg/g Fraction)	Octanol/Water
Luteolin	139.19 ± 2.84 ^a^	2.53
Baicalein	70.09 ± 2.32 ^b^	3.59
Apigenin	39.51 ± 1.60 ^c^	3.02
Kaempferol	23.69 ± 1.51 ^d^	3.11
Quercetin	16.06 ± 0.79 ^e^	1.82
Rosmarinic acid	9.97 ± 0.47 ^f^	1.82
Rutin	5.22 ± 0.39 ^g^	−1.13
Caffeic acid	ND	1.15
Gallic acid	ND	0.7

Means with different letters (a, b, c, d, e, f, and g) are significantly different (*p* < 0.05). ND = not detectable.

## Data Availability

Not applicable.

## Sample Availability

Samples of the luteolin- and baicalein-rich perilla leaf hexane fraction (PLH) are not available from the authors.
